# Intoxications involving methoxyacetylfentanyl and U-47700: a study of 3 polydrug fatalities

**DOI:** 10.1007/s00414-024-03263-7

**Published:** 2024-06-03

**Authors:** Arianna Giorgetti, Marcus Große Perdekamp, Giorgia Franchetti, Rebecca Pircher, Stefan Pollak, Susi Pelotti, Volker Auwärter

**Affiliations:** 1 Department of Medical and Surgical Sciences, Unit of Legal Medicine, University of Bologna, Via Irnerio 49, 40126 Bologna, Italy; 2 Institute of Forensic Medicine, Forensic Toxicology, Medical Center-University of Freiburg, Faculty of Medicine, University of Freiburg, Freiburg, Germany; 3 Legal Medicine, Department of Cardiac, Thoracic, Vascular Sciences and Public Health, University of Padova, Via Falloppio 50, 35121 Padua, Italy

**Keywords:** Forensic toxicology, NPS, Standard addition, Synthetic opioids, Toxicological significance score

## Abstract

Novel synthetic opioids (NSOs) represent an emerging group of novel psychoactive substances, acting as agonists at the opioid receptors. NSOs include fentanyl-related compounds, e.g. methoxyacetylfentanyl (MeACF), and non-fentanyl analogs, e.g. “U compounds” including U-47700. Here we present three cases of death involving MeACF and U-47700, with particular reference to preliminary data on pharmacokinetics and tissue distribution.

After a complete post-mortem examination, general unknown screenings and analysis of drugs of abuse were performed on postmortem samples by immunoassays, gas chromatography–mass spectrometry and liquid chromatography–mass spectrometry. To quantify the analytes of interest in post-mortem blood and tissues, the standard addition method was used. A toxicological significance score (TSS), weighing the role of the NSO in each death case, was assigned.

Case 1 died at the hospital after consumption of U-47700, methadone (serum levels: 2,600 ng/ml and 37 ng/ml), tilidine and benzodiazepines. In case 2, U-47700 (204 ng/ml) together with methadone (290 ng/ml), flubromazepam (480 ng/ml) and diazepam (300 ng/ml) were detected in peripheral blood. In case 3, methoxyacetylfentanyl (266 ng/ml), furanylfentanyl (4.3 ng/ml) 4-ANPP (15 ng/ml) and alprazolam (69 ng/ml) were quantified in femoral blood. In all cases, the NSO likely contributed to the death (TSS = 3).

NSOs appear to be often consumed in the setting of polydrug intoxications, especially in combination with other opioids and benzodiazepines, which often exert synergistic effects. The standard addition method remains the most reliable in post-mortem analysis and toxicological results should always be evaluated together with circumstantial and autopsy data.

## Introduction

On the market for new psychoactive substances (NPS), new opioids represent one of the most rapidly expanding groups, consisting of 74 compounds so far tracked by the European Monitoring Centre for Drugs and Drug Addiction (EMCDDA) [1]. These compounds, commonly called ‘new synthetic opioids’ (NSOs), bind to the µ-, δ-, and/or κ-opioid receptors in the peripheral and central nervous system (CNS), mimicking the effects of the classical opioids, but often with higher potency.

Since 2009, new NSOs introduced on the market mostly consisted of fentanyl and fentanyl derivatives [1], such as α-methylfentanyl and 3-methylfentanyl, often sold to heroin users [2]. Around 2019, in response to national and international controls and regulations [3, 4], the market shifted to benzimidazoles and other new opioids (e.g. “nitazenes”) [5–7], so that all new NSOs that appeared since 2021 were structurally distinct from fentanyl [1].

In the United States, NSOs spread rapidly and have been related to one of three waves of opioid overdose death epidemics, which was of such vehemence to be declared a public health emergency around 2017 [8]. In Europe, although opioids were estimated to be present in about three quarters of fatal overdoses, classical opioids (primarily heroin and substitution drugs like methadone) still play a major role in drug-related deaths [1]. However, outbreaks of deaths due to NSOs have been reported, and preliminary data showed a doubling in Europe from 2021 (n = 39) to 2022 (n = 79) [1]. NSOs pose a serious health threat and challenges for forensic toxicology, due to their in some cases extremely high potency and due to the fact that they are usually not detected by routine drug tests [9–11]. The wide availability, the circulation of multiple substances and the complex pattern of consumption represent further matters of concern [12].

One of the most prevalent non-fentanyl NSOs was U-47700 (3,4-dichloro-*N*-[(1R,2R)-2-(dimethylamino)cyclohexyl]-*N*-methylbenzamide) [3, 13], pertaining to a sub-class called “U compounds”, which was included among the Schedule I substances [14]. In Europe, U-47700 appeared around 2014 [3] and several death cases involving this compound have been reported in the literature since then [15–18].

Methoxyacetylfentanyl (2-methoxy-*N*-phenyl-*N*-[1-(2-phenylethyl) piperidin-4-yl]acetamide or MeACF) is a NSO structurally related to fentanyl and characterized by a 2-methoxyacetamide group replacing the propionamide moiety [19, 20]. It was synthesized around 1986 as an analgesic agent but never developed as a pharmaceutical drug [21]. After the scheduling of U-47700 in the United States, MeACF started circulating and the first seizures were reported in Europe around 2016. The compound was then associated to at least 13 deaths reported to the EMCDDA in 2016–2018 [20].

The identification of the role of NSOs in deaths is challenging, especially in the setting of polydrug toxicity, that dominates among drug-related fatalities according to the EMCDDA, and requires further research [1].

Here we report three cases of fatal polydrug intoxication involving U-47700 and MeACF in combination with other opioids and benzodiazepines. In each case, circumstantial, post-mortem and toxicological data are presented with preliminary considerations on pharmacokinetics (in case #1) and on the analysis of biological tissues by standard addition method (in case #2 and #3).

### Case #1

A 31 year-old man was found in a state of unconsciousness lying on his bed at around 21:30. A white powder, later tested positive for U47700, and further substances were found in his room. Cardiopulmonary resuscitation (CPR) was immediately attempted and the man was brought to the hospital at around 22:52. After restoration of the circulation, naloxone was administered and an artificial coma (low temperature, intubation) was induced. Laboratory examinations performed at hospital arrival showed anemia, increased biomarkers of inflammation (leukocytosis and elevated C reactive protein), elevated liver and pancreas enzymes (alanine and aspartate amino transferase 1397 and 906 U/l, alkaline phosphatase 170 U/l, gamma-glutamyl transferase 128 U/l, lactate dehydrogenase 1841 U/l, amylase 97 U/l), high creatinine levels (1.20 mg/dl), as well as elevated cardiac markers (creatine kinase-MB 192 U/l, myoglobin 831 ng/ml, troponin T 0.19 ng/ml, pro-brain natriuretic peptide 534 pg/ml). Serum samples were collected on the following days, and particularly on the first and second days of hospitalization, together with urines. Despite intensive care, life-preserving measures were discontinued 7 days later. The man suffered from hepatitis C and was known for a past history of narcotic drug abuse.

### Case #2

A woman called the police claiming that her 31 year-old boyfriend had become aggressive, throwing objects at her and hitting her, after starting to drink alcohol early in the morning. She left the apartment to call the police and on her return the man, who had a pocket knife in hands, claimed he didn’t want to survive anymore and he wanted to stab a policeman in order to be killed with a gunshot (so-called “suicide by cops”). She left again and, at the arrival of the law enforcement officers in the early afternoon, the man was found dead in his bed, with a syringe near his body on the mattress.

The man had a history of psychiatric diseases, previous suicide attempts by heroin intake, and was allegedly under methadone treatment. Multiple prescription drugs were found at home, including antidepressant drugs and bottles of alcoholic beverages.

### Case #3

**Fig. 1:**
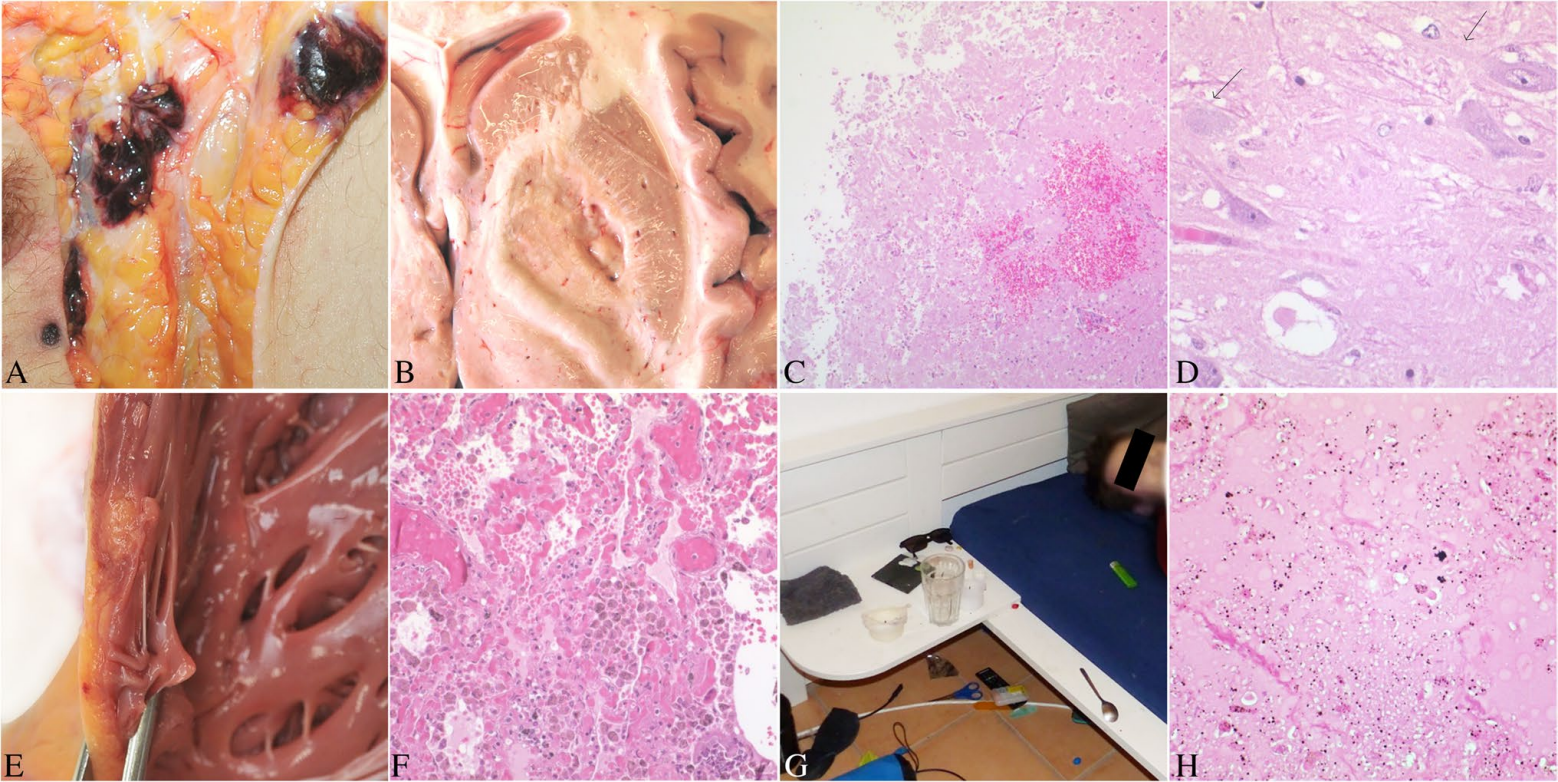
Case #1: A-D. **A**: fresh injection mark at the left groin. **B**: macroscopical area of encephalomalacia in the globus pallidus. **C**: histological appearance of the globus pallidus with hemorrhage and ischemical damage. **D**: spongy degeneration of the white matter. Case #2: E–F. **E**: fatty infiltration of the right ventricle. **F**: endoalveolar hemorrhage and edema in the lungs. Case #3: G-H. **G**: paraphernalia at the death scene investigation. **H**: putrefactive changes and endoalveolar edema in the lungs

A 20 year-old man was found dead in his bed. In his room, a mirror, a small plastic bag containing a white powder and other paraphernalia were found (Fig. 1), as well as empty boxes characteristic of online purchases, including some from United Kingdom. A piece of paper had written “furanylfentanyl” on it. On the monitor of his computer, a handwritten note read “I am sorry”.

The man had a history of narcotics and LSD consumption, used to self-experiment with drugs purchased online and was lastly seen in the late afternoon of the day before he was found dead, when he was heard “snoring loudly”.

## Materials and methods

### Reference materials and chemicals

Reference standards (RSs) of U-47700 and MeACF, and internal standards (ISs) (U-47700-D6 and fentanyl-D5) were obtained from Cayman Chemical (Ann Arbor, MI, USA). Formic acid (Rotipuran® ≥ 98%, p.a.), sodium hydroxide (≥ 99%, p.a., pellets), and potassium hydrogen phosphate (≥ 99%, p.a.) were purchased from Carl Roth (Karlsruhe, Germany). Methanol (HiPerSolv CHROMANORM®) was obtained from VWR Chemicals (Darmstadt, Germany); acetonitrile (ACN) (LC–MS grade) and ammonium formate 10 M (99.995%) were bought from Sigma Aldrich (Steinheim, Germany). Deionized water was prepared using a Medica® Pro deionizer from ELGA (Celle, Germany).

Mobile phase A (1% ACN, 0.1% HCOOH, and 2 mM NH_4_^+^ HCOO^−^ in water), mobile phase B (0.1% HCOOH and 2 mM NH_4_^+^ HCOO^−^ in ACN) and working solutions with the RSs in methanol were freshly prepared prior to analysis.

### Postmortem examination and sampling

Postmortem examinations were performed 2–5 days after the death at the Institute of Forensic Medicine of the University of Freiburg, including complete external examination, internal section of organs and sampling of biological material. Samples of tissues were fixed in formalin, dyed with standard hematoxylin and eosin (H&E) and then analyzed by optical microscope. Biological tissues and fluids were submitted to toxicological analyses. For case 1, serum and urine samples collected from the emergency department were also submitted to toxicological analyses.

### Toxicological analyses

‘General unknown screenings’ and tentative quantification of classical drugs of abuse were performed by means of CEDIA and DRI® immunoassays, gas chromatography (GC) with flame-ionization detection (FID), GC–mass spectrometry (GC–MS), and liquid chromatography with tandem mass spectrometry (LC–MS/MS) using validated methods, accredited under ISO/IEC 17025 for forensic purposes [22, 23]. Antemortem serum samples and urine samples (after enzymatic hydrolysis using ß-glucuronidase) were analyzed by means of LC–MS/MS using previously published and validated methods, after update and revalidation [17, 22, 23].

A standard addition approach was used for the quantification of U-47700 and MeACF in fluids and tissue samples taken at the postmortem examination (case #2 and #3). This method has the potential to overcome the matrix effect expected in postmortem materials.

A previously employed procedure was applied [22]. Briefly, 0.5 g or 0.5 ml of material (central and peripheral blood, liver, kidney, brain and stomach content) was measured, after mincing with clean surgical scissors in the case of tissues. The material was placed in a 1.5 ml plastic tube with a cap containing 1 ml phosphate buffer pH 6; the tube was capped, put into a homogenizer (Beads Crusher lT-12; TAITEC, Koshigaya, Japan), and vigorously shaken at maximum speed (3300 r/min) for 3 min. The homogenates were further diluted with 9 ml of phosphate buffer pH 6 (resulting in 0.5 g in 10 ml) and stored 24 h at -20 °C prior to extraction.

Aliquots consisting of 100 μl supernatant of homogenated specimen, containing an unknown amount of the analyte of interest, were spiked with 10 μl of IS (either U-47700-*D*_*6*_ or fentanyl-*D*_*5*_) and varying volumes of RS solution, to achieve a six-point-calibration curve. To receive concentrations in the range of the calibration, 1:10; 1:20 or 1:100 dilutions of the homogenates in phosphate buffer pH 6 were analyzed.

Resulting tissue calibrators were, for case 2: central, peripheral blood, brain, liver, kidney: 0, 10, 50, 100, 250, 500 ng/ml; stomach content: 0, 20, 100, 200, 500, 1000 ng/ml.

For case 3: peripheral blood, liver and kidney: 0, 10, 50, 100, 250, 500 ng/ml; brain, and central blood: 0, 100, 500, 1000, 2500, 5000 ng/ml; stomach content: 0, 1000, 5000, 10,000, 25,000, 50,000 ng/ml.

Sample preparation consisted of a protein precipitation step as described by Müller et al. [24]. Briefly, after fortification with IS and spiking with RS, 100 µl ammonium formate (10 M) and 1 ml ice-cold acetonitrile (-20 °C) were added. The sample was shaken, centrifuged for 10 min at 2,900 g and the supernatant, transferred to a separate vial, was evaporated under a gentle stream of nitrogen (40 °C). The dry residue was reconstituted in 100 µl mobile phase (A:B 95:5, v/v).

Analyses were performed on a Shimadzu Nexera X2 LC-30AD (Duisburg, Germany) coupled to a QTRAP 5500 triple quadrupole linear ion trap instrument (Sciex, Darmstadt, Germany). The LC system was equipped with a Kinetex® F5 column (2.6 µm, 100 × 2.1 mm) (Phenomenex, Aschaffenburg, Germany) with a corresponding pre-column (2.1 mm) from Phenomenex. Autosampler temperature was maintained at 10 °C and the injection volume was 10 μL. Chromatographic conditions were as follows (flow 0.5 ml/min): an initial hold at 5% B for 1 min, increased to 22.5% at 4.5 min, then to 32.5% at 10.75 min, then to 95% at 13.5 min, holding at 95% for 2 min and then decreasing to initial conditions in 0.5 min, held for 3.5 min. Total runtime was 19.5 min. Samples were analyzed in ESI positive mode with an ion spray voltage of 4,500 V. The gas settings were as follows: curtain gas 40 psi, collision gas medium, ion source gas (1) 60 psi, ion source gas (2) 70 psi. Source temperature was set to 500 °C.

For each specimen, the procedure was repeated 5 times on 5 different days, to check for interday repeatability and the relative standard deviation (RSD%) was calculated.

### Toxicological significance score

In each case, after careful evaluation of all data, the NSO of interest was assigned a Toxicological Significance Score (TSS), according to the publication by Elliott et al. [25]. The TSS is an instrument developed to help assess the role of an NPS in a fatality on the basis of various factors (presence of the compound and/or metabolites, nature and concentrations of substances, death circumstances, postmortem findings, etc.). According to the score, the role in a fatality can range from ‘1’, i.e., “alternative cause of death”, to ‘3’, i.e., “… likely to have contributed to toxicity/death, even in presence of other drugs”.

## Results

### Post-mortem examination

#### Case #1

The man exhibited a poor dental and hygiene status, as well as multiple scars in the groin regions and on the corresponding femoral veins. An investigation of the typical areas for injection marks was performed and revealed a relatively fresh injection mark in the left groin and in the corresponding left femoral vein, surrounded by bleeding in the subcutaneous tissues (Fig. 1A). Signs of iatrogenic injection marks and CPR were also noted. The brain displayed severe edema (1,585 g), with herniation of the cerebellar tonsils. Basal ganglia showed a small oval area of brownish tissue softening, referred to as encephalomalacia, at the right globus pallidus (Fig. 1B). Signs of aspiration were noted in the airways; lungs were overinflated and edematous (1,475 g together). The heart (415 g) and the liver (2,165 g) showed moderate and severe enlargement, respectively. Kidneys were congested, with barely distinguishable corticomedullary differentiation.

Histology demonstrated necrosis, reactive hyperplasia of the vasal endothelium and hemorrhagic extravasation in the area of encephalomalacia. Moreover, a spongy degeneration of the myelin and of the gray matter was noted, as indicative of recent ischemic damage (Fig. 1C-D). Histology of the lungs confirmed macroscopic findings with emphysema, endoalveolar hemorrhage and desquamation of macrophages in the alveoli, together with signs of acute stasis.

#### Case #2

The external examination of the man revealed multiple scars on both wrists and forearms. Just below the crook of the left elbow, within a small blue-grey hematoma, a point-like injection mark was noted, and confirmed by sectioning the skin revealing a subcutaneous bleeding.

The brain (1,634 g) exhibited severe edema with flattening of the sulci and compression of the ventricles. The airways contained small particles of food with an acidic pH. The lungs (1,580 g) were swollen showing peripheral pulmonary hyperinflation. The heart (390 g) showed fatty degeneration in the right ventricle (Fig. 1E), while the left ventricle was slightly dilated with areas of fibrosis, each measuring a maximum of 5 mm of diameter. The increase in connective tissue was confirmed under UV lighting. The stomach contained 150 ml of dark-brown fluid with small vegetable particles. The liver appeared enlarged (2,143 g) and fatty, and the kidneys congested. The bladder was filled with 350 ml of urine.

Histology confirmed macroscopic findings and particularly showed endoalveolar edema and hemorrhage in the lungs with multiple signs of acute vascular and capillary stasis (Fig. 1F). No myocardial atrophy, necrosis or inflammation was noted in the area of fatty infiltration.

#### Case #3

External examination was unremarkable, except for some stains of reddish-brown liquid material on the face, at the nasal openings and in the mouth. At the internal section, the brain (1,689 g) showed a pronounced watery swelling with flattening of sulci. In the airways, red-brownish liquid with fine particulate matter and acid pH was found, as in the esophagus. Lungs (935 g together) were overinflated and showed edema, with spillage of foamy red-brownish liquid at the cut. The stomach contained 200 ml of brown liquid with tiny food components. Other organs were unremarkable (heart: 292 g, liver: 1,403 g). Histology confirmed macroscopic findings, with diffuse endoalveolar edema and hemorrhage and multiple endoalveolar macrophages (Fig. 1H).

### Toxicological results

#### Case #1

Toxicological analysis of blood and urine collected during the hospitalization showed the presence of U-47700, methadone and its metabolite EDDP, tilidine and nortilidine, nordazepam, clonazepam and 7-aminoclonazepam, as well as Δ-9-tetrahydrocannabinol (THC) and its metabolites. Table 1 shows the concentrations of the analytes found in ante-mortem and post-mortem samples. The initial concentration of U-47700 decreased from 2,600 ng/ml to 1,300 ng/ml within 5.5 h. Urine analysis confirmed the intake of U-47700 by showing the presence of its main metabolites *N*-desmethyl-U-47700 and *N*,*N*-didesmethyl-U-47700. Given the very low levels of U-47700 in post-mortem blood and the little volume of material available, no quantification by standard addition method was performed.

**Table 1:** PM: post-mortem. EDDP: 2-Ethylidene-1,5-dimethyl-3,3-diphenylpyrrolidine; THC: Δ-9-Tetrahydrocannabinol; THC-COOH: 11-Nor-9-carboxy-Δ^9^-tetrahydrocannabinol; 11-OH-THC: 11-Hydroxy-Δ9-tetrahydrocannabinol; LOQ: limit of quantification: 5 ng/ml for diazepam, midazolam, nordazepam and sufentanil, 1 ng/ml for U-47700. *: detected but not quantified

Day 1-serum [ng/ml] h 23.00		Day 2-serum [ng/ml] h 04.30		Day 9-PM blood [ng/ml]	
U-47700 Methadone EDDP Tilidine Nortilidine Nordazepam Clonazepam 7-Aminoclonazepam THC THC-COOH 11-OH-THC	2,600 37 0.8 2.4 23 < LOQ 32 55 1.7 136 1.2	U-47700 Methadone EDDP Tilidine Nortilidine Nordazepam Clonazepam 7-Aminoclonazepam Midazolam Alpha-Hydroxymidazolam	1,300 20 0.7 0.5 8.0 < LOQ 49 62 65 40	U-47700 Methadone EDDP Morphine Sufentanil Diazepam Nordazepam Midazolam Hydroxymidazolam 7-Aminoclonazepam	2.1 5 3.9 170 9.1 < LOQ < LOQ < LOQ 17 4
		Day 2-urine [ng/ml]		Day 9-PM urine [ng/ml]	
		U-47700 OH-U47700 N-desmethyl-U-47700 N,N-didesmethyl-U-47700 Tilidine Nortilidine Nordazepam Clonazepam Midazolam Hydroxymidazolam 7-Aminoclonazepam Naloxone Lidocaine Noscapine Oxazepam THC-COOH	720 * * * * * < LOQ 29 16 68 104 170 58 0.7 20 *	U-47700 Methadone EDDP Morphine Diazepam Nordazepam Temazepam Oxazepam Midazolam Hydroxymidazolam 7-Aminoclonazepam Sufentanil	< LOQ 3.3 2.6 520 5 7.2 5.4 25 2 17 4 < LOQ

#### Case #2

LC–MS/MS analyses of femoral blood allowed to identify U-47700 (220 ng/ml), methadone (290 ng/ml), EDDP (14 ng/mL), flubromazepam (480 ng/ml) and hydroxyflubromazepam (85 ng/ml), diazepam (300 ng/ml), nordazepam (100 ng/ml), temazepam (10 ng/ml), oxazepam and duloxetine (< limit of quantification (LOQ), each 10 ng/ml) as well as ethanol (1.64 g/kg).

Methadone, EDDP, diazepam and metabolites as well as duloxetine and metabolites were qualitatively confirmed in urine, where ethanol was quantified at 2.33 g/l.

Results of the standard addition method on fluids and tissues are shown in Table 2.

**Table 2:** Post-mortem concentrations of U-47700 (case #2) and methoxyacetylfentanyl (MeACF)(case #3) as determined using the standard addition method (except for urine samples, which were measured using validated methods). RSD: relative standard deviation. For stomach content * = levels refer to relative concentrations and not absolute values. The volume of the stomach content was 150 ml for case #2 and 200 ml for case #3

	Case #2			Case #3		
Specimen	U-47700 concentrations [ng/g or ng/ml]	Equation (R^2^ coefficient correlation)	RSD (%)	MeACFconcentrations [ng/g or ng/ml]	Equation (R^2^ coefficient correlation)	RSD (%)
Femoral blood	204 ± 17	y = 0.001x + 0.128 (0.999)	9.34	266 ± 16	y = 0.097x + 12.24 (0.998)	5.95
Central blood	470 ± 31	y = 0.001x + 0.337 (0.999)	6.5	450 ± 22	y = 0.009x + 2.24 (0.999)	4.91
Brain	278 ± 14.2	y = 0.002x + 0.209 (0.999)	5.1	1,500 ± 126	y = 0.011x + 7.35 (0.996)	8.53
Liver	198 ± 18.9	y = 0.002x + 0.148 (0.999)	9.6	85 ± 6.2	y = 0.110x + 5.41 (0.998)	6.52
Stomach content *	1,500 ± 81	y = 0.001x + 0.602 (0.999)	5.6	27,000 ± 320	y = 0.001x + 12.53 (0.998)	1.19
Kidney	293 ± 20	y = 0.002x + 0.223 (0.999)	6.89	492 ± 23	y = 0.097x + 23.77 (0.997)	4.68

#### Case #3

Toxicological analyses of case 3 allowed to identify the following compounds by LC–MS/MS analysis of femoral blood: MeACF (340 ng/ml), furanylfentanyl (4.3 ng/ml) 4-ANPP (15 ng/ml), alprazolam (69 ng/ml) and alpha-hydroxyalprazolam (3.2 ng/ml) as well as traces of diazepam and nordazepam (< LOQ of 5 ng/ml). The same substances were identified in heart blood. In urine, the drugs detected in blood were confirmed and MeACF was quantified (282 ng/ml).

Results of the standard addition method on fluids and tissues are shown in Table 2, together with equations and RSD% values.

## Discussion

Typical pharmacological effects of opioids and NSOs, which also present activity at the μ-opioid receptor in the CNS, include miosis, sedation, bradycardia, hypothermia, constipation and dose-dependent respiratory depression, with the risk of fatal overdoses [26]. In intoxications involving opioids and NSOs, post-mortem findings are usually nonspecific. According to the literature [18, 27], all cases here reported did not display anatomopathological causes of death, but only signs of agonal aspiration of gastric content, which suggest the absence of neurological reflexes due to neurological impairment, as well as pulmonary and brain edema of various grades.

In case #1, protrusion of the cerebellar tonsils (a sign of severe cerebral swelling) was accompanied by unilateral necrosis of the globus pallidus. Lesions of the basal ganglia are usually bilateral and could be due to various causes, like e.g. carbon monoxide poisoning, heart disease, asphyxia, head trauma or drug overdose [28, 29]. Bilateral necrosis at the globus pallidus was detected in association with oxycodone, methadone, heroin and morphine [28, 30–32], so that it was suggested as a diagnostic clue of opioid intoxication [30]. Due to poor vascularization, the globus pallidus appears especially susceptible to cerebral hypoxia–ischemia. Recurrent episodes of hypoxia might be the primary cause for the necrosis, though the possibility of a direct neuronal toxicity has been discussed [32, 33]. To the best of our knowledge, this is the first unilateral globus pallidus injury found in association with a NSO consumption, even though other factors (like hypoglycemia or CPR) might also be responsible for this finding.

A wide range of concentrations has been found in fatalities related to U-47700, with sometimes very high levels of up to 2,100 ng/ml [15, 18]. The concentrations detected in case #1 (2,600 ng/ml) are among the highest so far reported, likely due to the recent consumption of a very large dose possibly combined with impaired clearance. The consumption of a large amount of U-47700 by intravenous injection, as demonstrated by the recent injection mark shown at the post-mortem examination and the extraordinarily high serum concentration, could be explained by tolerance developed after continued opioid misuse. Indeed, the man had a history of intravenous drug use and hepatitis C, which are consistent with post-mortem findings of multiple scars and a poor dental hygiene. Moreover, U-47700 was described by users for its short-lasting euphoric effects that could prompt the consumption of multiple increasing doses [13] and this might have occurred in the present case.

Given the availability of samples collected at the hospital, it was possible to estimate that U-47700 decreased to half of the initial concentration within 5.5 h, showing a serum to urine ratio of 2:1. An elimination half-life of 5.5 h is close to the finding in another case-report, where a half-life of 6 h was reported [17]. Nevertheless, poor hepatic and renal function, as shown by blood test and by post-mortem findings (signs of chronic kidney failure) might have influenced the elimination in our case. For what concerns the detection of U-47700 in post-mortem blood 9 days after the consumption (considering the 7 days of hospitalization + 2 days post-mortem interval), poor clearance, enterohepatic circulation and/or accumulation in tissues, e.g. fat tissue, as observed in pig models [34], with later redistribution to the vascular compartments are conceivable. However, the storage of the body in a refrigerated environment could also have prolonged the detection time of the NSO, since U-47700 appears rather stable up to 10 days under refrigeration [35].

The other drugs detected at the arrival to the hospital, particularly methadone, tilidine, nordazepam, clonazepam and their metabolites, were within or even below the therapeutical levels reported in the literature, although these tipically refer to serum/plasma [36]. Although these drugs, including THC, share CNS depressant effects, their role in the fatality can be considered minor. However, their detection suggests that NSOs tend to be consumed in combination with other opioids (methadone and tilidine) and benzodiazepines, as shown in other cases [35, 37–39]. Additional substances detected, namely naloxone, morphine, midazolam and sufentanil, were likely administered in the hospital setting. The extremely high level of U-47700 in the first clinical sample appears highly indicative of a fatal effect of the drug, justifying, together with the evaluation of circumstantial, clinical, and post-mortem findings, the assignment of a TSS of 3 to U-47700, in the setting of a combined intake of multiple drugs.

In this case it could be hypothesized that the intoxication caused primarily a CNS depression, which in turn led to a cardiac and multiorgan insufficiency, as shown by laboratory exams. The pulmonary findings might be related to a depression of the respiratory drive, followed by the aspiration of gastric material and hospitalization with CPR and mechanical ventilation, resulting in a mixed pattern of edema and emphysema.

A similar combination of drugs was detected in case #2, where U-47700 was accompanied by ethanol, methadone, duloxetine and benzodiazepines, particularly flubromazepam, and diazepam as well as its metabolites nordazepam, temazepam, and oxazepam.

The deceased had no relevant pathological findings at the autopsy, except for a fatty degeneration of the right ventricle, which is sometimes observed after chronic alcohol abuse, and could be a sign of arrhythmogenic right ventricular cardiomyopathy (ARVC), a cause of sudden cardiac death [40]. However, fatty infiltration with no myocardial atrophy, necrosis or inflammation (which are typical of ARVC) is commonly found in non-cardiac deaths [40] and the identification of severe brain edema suggests a relatively slow death, as described with opioid intoxication, rather than a sudden occurrence.

It is well known that therapeutic, toxic and fatal levels of opioids, and particularly methadone, tend to overlap [36] due to multiple factors including the development of tolerance [41]. In case #2, the circumstantial data of methadone treatment for opioids dependency matched the toxicological analysis and suggested a state of tolerance in the setting of a therapeutical consumption. Therefore, the role of co-consumed substances and U-47700 became of particular interest. The compound shows a lower potency compared to many other NSOs, but still appears to be about 10 times more potent in vivo than morphine [11]. In a series of 16 fatal (mono or mixed) U-47700-related intoxications, death was reported with concentration starting from 17 ng/ml with a median level of 247 ng/ml, which is similar to the level displayed by the deceased (204 ng/ml) in femoral blood [35]. Lower levels were detected in a mono-intoxication case by McIntyre et al. (190 ng/ml) [42] and in several cases of polydrug consumption, particularly when flubromazepam was detected [18]. The here-in presented combination of U-47700 with flubromazepam (480 ng/ml) further confirms the need to screen for designer benzodiazepines and other NPS groups beside NSOs [17, 18, 43]. Designer benzodiazepines are thought to have a synergistic effect on opioid-induced respiratory depression, as shown for traditional benzodiazepines and for ethanol (1.64 g/kg), which were both detected in case #2 [18, 44, 45]. The agitation and delirium manifested by the victim are not common in the toxidrome of opioids or designer benzodiazepines [45], but they have been reported as atypical symptoms of flubromazepam intake [46, 47] and could possibly be explained by the elevated ethanol level. Indeed, ethanol concentrations in blood and urine are consistent with acute toxicological effects after complete absorption and distribution at the time of death [48]. Other substances were either in their therapeutical range or considered metabolites [36]. Given the severe brain and pulmonary edema revealed at the post-mortem examination and the results of the toxicological analyses, the death was deemed a fatal polydrug intoxication caused by several CNS depressant agents, particularly U-47700 (TSS = 3), methadone, flubromazepam and ethanol.

A few cases on the distribution of U-47700 in tissues are available in the literature [18, 49, 50]. The whole content of the stomach (150 ml) was not analyzed, so it is possible to provide only a relative concentration.

In 26 fatalities associated with U-47700, the higher relative stomach concentrations compared to blood levels were considered suggestive of an oral intake [18].

However, in our case a syringe was found near the body and a fresh injection mark was noted at the post-mortem examination, as for injection. Studies on morphine suggest that, due to entero-hepatic circulation and reflux of duodenal contents back into the stomach, morphine levels in blood and stomach cannot be used to determine the site of administration [51]. A entero- hepatic circulation was also conceived for U-47700 due to bile and duodenum high levels in pigs following intravenous administration [34].

Nevertheless, due to its pKa, U-47700 is rapidly trapped in the acid medium of stomach contents and the (numerous) blood vessels of the stomach wall undergo autolysis already at an early stage of the postmortem interval.

Higher concentrations were detected in central blood (C) than in peripheral blood (P), with a C/P ratio of 2.3. This is consistent with a series of 26 fatalities, in which U-47700 showed a moderate propensity for post-mortem redistribution with a C/P ratio in the range of 1–8 [18]. However, several additional factors including dose, route of administration and post-mortem interval should be considered in order to assess a drug’s propensity for redistribution [52]. In case #2, the higher C concentration might be due to a concentration-gradient driven post-mortem diffusion of the drug from the stomach.

In case #3, the presence of multiple boxes and packages of drugs purchased online represented a strong hint towards the consumption of NSOs, given that internet shops are a primary source for obtaining NPS [53]. Moreover, no label reporting the presence of MeACF was noted, so that the accidental consumption of mislabeled powder seems possible.

Although lungs weighed less compared to similar cases of opioids intoxication [54], including cases #1 and #2, brain edema, and froth, a proxy for pulmonary edema, were detected.

Toxicological analyses allowed to identify a cocktail of substances, particularly MeACF, furanylfentanyl, 4-ANPP, alprazolam and its metabolite, as well as traces of other benzodiazepines. According to the data so far available, MeACF and furanylfentanyl should have a pharmacological potency comparable to fentanyl [55, 56]. Particularly, past in vitro and in vivo studies have considered that MeACF is around 3 to 5 times less potent than fentanyl [54]. However, median blood levels in intoxication cases were relatively low: 15.1 ng/ml in a series of 11 deceased described by Fogarty et al. [21] and 34 ng/g among other 11 fatalities, with the highest reported concentration of 140 ng/g in a case of monointoxication [54]. Thus, the authors concluded that MeACF carries a high risk for accidental intoxication [54]. The levels here reported are well above the ones described by other authors [21, 54], suggesting that these are consistent with a severe CNS depressant effect. The note left on the computer might additionally suggest a massive consumption of drugs with suicidal intention. Moreover, an additive worsening of the respiratory depression can be assumed in the case of co-consumption of other potent opioids, as in the present case for furanylfentanyl, and of benzodiazepines [56]. Comatose-fatal effects of furanylfentanyl were described in 17 cases of death with blood concentrations in the range 0.2–2.7 ng/ml [36, 56]. On the basis of this data, the concentration of furanylfentanyl of our case appears in a range where strong side effects (sedation, hypotension, respiratory depression) can be anticipated without the existence of an acquired opioid tolerance.

4-ANPP could represent a synthesis by-product or a metabolite of furanyfentanyl. In both cases, the compound is thought to have some biological activity, but much lower than that of morphine [56] and did likely not play a major role in the present fatality.

Alprazolam levels were slightly above the therapeutical range described by Schulz et al. [36], although multiple factors, including metabolization and redistribution, might affect the reliability of postmortem blood levels. The compound is commonly encountered in fatal polydrug intoxications and could amplify the respiratory depression induced by co-consumed drugs [57].

Although the subject was used to try drugs purchased online, and might have developed a tolerance to opioids, given the co-consumption of highly potent NSOs with supra-therapeutical (alprazolam) and therapeutical (diazepam) levels of prescription benzodiazepines and the signs of brain edema, the death was explained as a consequence of a mixed intoxication, with MeACF (TSS = 3), furanylfentanyl and alprazolam playing a major role.

To the best of our knowledge, this is the first time that concentrations of MeACF in multiple tissues are reported, beside blood and brain levels [58]. In a previous case series, brain concentrations of MeACF were 3 times higher than those retrieved in blood (0.074 mg/kg vs 0.022 mg/kg) [58]. This was also seen in our case, with fivefold higher brain concentrations compared to blood. The high concentration in the brain further confirms the hypothesized mechanism of fatal CNS depression. Moreover, together with the lower levels in the liver and in urine, it is suggested that the compound was not extensively metabolized at the time of death and led to a rather acute death. Very high relative concentrations were estimated in the gastric content (Table 2) and this might be due to an oral ingestion or insufflation (followed by swallowing) of the compound. As already shown for case #2, MeACF levels were higher in central than in peripheral blood, although only by a factor of 1.7, and this might be explained by the route of intake.

The cases here presented had several factors in common. The deceased were healthy young men with a history of psychiatric diseases and/or past drug consumption, including a propensity for self-administering novel substances. Another rather constant finding is represented by drug powder residues (sometimes mislabeled) and paraphernalia at the death scene, consisting of a domestic environment. As expected, no specific post-mortem findings were discovered, although the necrosis of the globus pallidus might be related to the NSO consumption.

As emerged from the present series and from past literature [17, 18, 54], NSOs appear to be often consumed in a setting of polydrug use, especially in combination with other opioids (classical or NSOs) and benzodiazepines (including designer benzodiazepines). The co-consumption of multiple compounds further complicates the interpretation of the role of an NPS in the fatality, considering the possibility of synergistic effects. In our series, NSOs were always considered as a major contributor to the death (TSS = 3, i.e., “… likely to have contributed to toxicity/death, even in presence of other drugs” [25]), given the high potency described in the literature, high concentrations detected in blood, low levels of other drugs and the consistency of all data. In each postmortem case involving novel compounds and especially when the complexity is increased by polydrug consumption, a multidisciplinary evaluation of circumstantial, postmortem, histological and toxicological data is of paramount importance, and further information can be obtained, when enough material is available for analysis, by the distribution of drugs in tissues.

A limitation of the standard addition method, and of the present analysis, is represented by the fact that, considering only those data points with abscissa values equal to and greater than zero, nonlinearity might occur below the spiking level zero. However, blank addition could be combined with standard addition to overcome this limitation [59].

Although blood concentrations determined by LC–MS/MS analysis and by standard addition methods were approximately in the same range (220 ng/ml vs. 204 ng/ml for case #2, and 340 ng/ml vs 266 ng/ml for case #3), standard addition remains the most reliable quantification method that can overcome matrix effects [23] and can therefore be encouraged.
